# Natural diversity of lactococci in γ-aminobutyric acid (GABA) production and genetic and phenotypic determinants

**DOI:** 10.1186/s12934-023-02181-4

**Published:** 2023-09-09

**Authors:** Valérie Laroute, Nathalie Aubry, Marjorie Audonnet, Muriel Mercier-Bonin, Marie-Line Daveran-Mingot, Muriel Cocaign-Bousquet

**Affiliations:** 1 Toulouse Biotechnology Institute (TBI), Université de Toulouse, CNRS, INRAE, INSA, Toulouse, France; 2 Toxalim (Research Centre in Food Toxicology), Université de Toulouse, INRAE, ENVT, INP-Purpan, UPS, Toulouse, France

**Keywords:** *Lactococcus lactis*, *Lactococcus cremoris*, γ-aminobutyric acid, GAD system

## Abstract

**Background:**

γ-aminobutyric acid (GABA) is a bioactive compound produced by lactic acid bacteria (LAB). The diversity of GABA production in the *Lactococcus* genus is poorly understood. Genotypic and phenotypic approaches were therefore combined in this study to shed light on this diversity. A comparative genomic study was performed on the GAD-system genes (*gadR*, *gadC* and *gadB*) involved in GABA production in 36 lactococci including *L. lactis* and *L. cremoris* species. In addition, 132 *Lactococcus* strains were screened for GABA production in culture medium supplemented with 34 mM L-glutamic acid with or without NaCl (0.3 M).

**Results:**

Comparative analysis of the nucleotide sequence alignments revealed the same genetic organization of the GAD system in all strains except one, which has an insertion sequence element (IS*981*) into the P_*gadCB*_ promoter. This analysis also highlighted several deletions including a 3-bp deletion specific to the *cremoris* species located in the P_*gadR*_ promoter, and a second 39-bp deletion specific to *L. cremoris* strains with a cremoris phenotype. Phenotypic analysis revealed that GABA production varied widely, but it was higher in *L. lactis* species than in *L. cremoris*, with an exceptional GABA production of up to 14 and 24 mM in two *L. lactis* strains. Moreover, adding chloride increased GABA production in some *L. cremoris* and *L. lactis* strains by a factor of up to 16 and GAD activity correlated well with GABA production.

**Conclusions:**

This genomic analysis unambiguously characterized the cremoris phenotype of *L. cremoris* species and modified GadB and GadR proteins explain why the corresponding strains do not produce GABA. Finally, we found that glutamate decarboxylase activity revealing GadB protein amount, varied widely between the strains and correlated well with GABA production both with and without chloride. As this protein level is associated to gene expression, the regulation of GAD gene expression was identified as a major contributor to this diversity.

**Supplementary Information:**

The online version contains supplementary material available at 10.1186/s12934-023-02181-4.

## Background

Lactic acid bacteria (LAB) are widely used in the food industry (cheese, buttermilk, sour cream, yogurt) as acidifiers (converting sugars into lactic acid) [1], food thickeners [2] and as bacteriocin producers [3, 4]. They also contribute to the flavor of dairy products [5] and interest in their potential use as cell factories for the chemical industry (biofuels, solvents, bio-based plastics) has grown in recent years [6–9]. Another attractive feature of LAB is their ability to produce a range of molecules with healthcare applications such as bioactive peptides, vitamins, hyaluronic acid and γ-aminobutyric acid (GABA) [10–12]. GABA, the most widely distributed neurotransmitter in the sympathetic nervous system [13], has been a particular focus of research for several years because of its many health benefits. GABA plays a crucial role in lowering blood pressure, reduces the risk of lung adenocarcinoma, and has been shown to have anti-diabetic, neuroprotective, antidepressant, anti-inflammatory and visceral antinociceptive properties [14–16].

The most studied LAB genus to date is *Lactobacillus*, which is generally considered one of the best for GABA production [17–19]. However, *Lactococcus* species also produce GABA and interest in this genus has gradually increased in recent years. The genome of *Lactococcus lactis* NCDO2118, a non-dairy GABA-producing bacterium, was sequenced in 2014 [20], and in more recent studies, new lactococci with a high potential for GABA production have been isolated in fermented milk [21, 22] and in fermented fish products [23].

*Lactococcus* species are found in many ecological niches (milk, plants, digestive tract, etc.) but the two main species of interest in the dairy and nutraceutical industries (following a recent taxonomic reclassification of *Lactococcus lactis* into two distinct species) are *L. lactis* and *L. cremoris.* While these species were previously distinguished based on a handful of phenotypic characteristics, new molecular methods [24–27] have allowed a better discrimination of these two species, based on average nucleotide identity (ANI) and tetranucleotide frequency correlation coefficients (TETRA) [28]. The *cremoris* species is phenotypically heterogeneous with strains having the typical cremoris phenotype and others having a lactis phenotype [29, 30]. The cremoris phenotype is characterized by an inability to produce GABA [31], to hydrolyze arginine [32], to grow at 40 °C and in 4% (w/v) NaCl.

GABA is synthesized from glutamate by the enzyme glutamate decarboxylase (GAD). This enzyme participates in the control of the acidification of the cytosolic environment by decarboxylating glutamate (acid substrate) into a neutral compound (GABA) by consuming H + ions, thus effectively protecting cells from acid stress [33, 34]. The glutamate decarboxylase gene is part of a GAD system involving a chloride-dependent *gadCB* operon (with *gadC* gene encoding glutamate/GABA antiporter and *gadB* gene encoding GAD) and *gadR* gene encoding positive regulator. This genetic organization was proposed in 1998 for a few strains [35, 36] and no further studies of the genetic organization of the GAD system in lactococci have since been published. It is unclear whether this genetic organization is common to all lactococci or just a few. Furthermore, the diversity of GABA production in lactococci has yet to have been studied in detail. Some *cremoris* strains have been described not to produce GABA [37] but is this a common feature of all *L. cremoris* strains with the cremoris phenotype? Two further interesting questions are whether and to what extent any *L. cremoris* species with the lactis phenotype and all *L. lactis* species produce GABA?

To shed some light on the genetic and phenotypic diversity of lactococci for GABA production, a set of 132 *Lactococcus* strains from different biotopes was screened for GABA production in semi-synthetic culture medium supplemented with 34 mM L-glutamic acid. The strains were also screened in the presence of chloride. The organisation of the genes involved in GABA production were also analysed and the nucleotide sequences of the genes were compared for 36 lactococci from the initial screening panel to determine whether this diversity in GABA production can be explained by the phenotypic and genotypic data obtained.

## Methods

### Organisms and growth conditions

The microorganisms used throughout this work were 88 *L. lactis* strains and 20 *L. cremoris* strains with the lactis phenotype and 24 *L. cremoris* strains with the cremoris phenotype from public and private collections. All bacterial strains are listed in Additional file 1 Table S1. The strains were stored at -80 °C in medium supplemented with glycerol (20%) in 96–deep-well plates (30 µL/well).

### Screening for GABA production

Overnight cultures (from deep well plates) in semi-synthetic medium (1 mL) containing glucose (20 g/L), yeast extract (YE) (10 g/L) KH_2_PO_4_ (9 g/L), K_2_HPO_4_ (7.5 g/L), MgSO_4_ 7 H_2_O (0.2 g/L), MnSO4 (0.05 g/L), grown at 30 °C without shaking, were inoculated (1:6) in 200 µL of fresh medium for 3 h (i.e. until the optical density at 580 nm, OD_580_, reached 0.6–0.8, corresponding to late-exponential phase cells). For GABA production screening, precultured cells were inoculated at 2.5% in new microplates containing 200 µL of the semi-synthetic medium supplemented with 34 mM L-glutamic acid (the precursor of GABA), with or without NaCl (0.3 M). The initial pH was 6.6. All experiments were performed in duplicate.

Biomass production was estimated by measuring OD_580_ directly in the microplates without sampling every 30 min for 24 h with a microplate reader (SpectraMax Plus, Molecular Devices). Samples (170 µL) were collected at 24 h in order to measure the GABA concentration and arginine consumption (methods described below).

### Growth rate measurements at 40 °C and with addition of 4% (w/v) NaCl at 30 °C

Cells were grown overnight as described above for GABA production measurements in the same culture medium. These precultures were then used to inoculate (at 2.5%) two sets of microplates: one containing 200 µL of the semi-synthetic medium incubated at 40 °C, the second containing 200 µL of the semi-synthetic medium with 4% (w/v) NaCl incubated at 30 °C. The initial pH was 6.6. All experiments were performed in duplicate.

### Flask cultures

To study GAD activity in the presence and absence of chloride, bacterial cells (NCDO2118, S642, EIP3I, NCDO2727, MG1363 strains) were grown under static conditions in flasks containing 500 mL semi-synthetic medium (described above) at 30 °C with and without chloride. Cells from overnight cultures were inoculated to obtain an initial OD_580_ of 0.25. Flask fermentation experiments were performed in duplicate.

Bacterial growth was monitored by measuring OD_580_ (Biochrom Libra S11, 1 one unit of absorbance equivalent to 0.3 g/L) on samples collected every 30 min. GAD activity was measured after 6 h in sample volumes containing 150 mg of cells. GABA concentrations were also measured at 6 and 24 h by HPLC.

### Growth rate estimates

Maximum growth rates (*µ*_max_) were calculated from four to five consecutive OD_580_ measurements at between 0.5 and 3.0 h of growth in microplates or flasks using the following formula: (*µ*_max_ = ∆lnOD_580_/∆t, where t is time).

### GABA concentration

GABA and arginine concentrations in culture supernatants were measured by HPLC (Agilent Technologies 1200 Series, Waldbronn, Germany) as previously described [38]. Briefly, proteins in the sample were precipitated by adding four volumes of methanol to one volume of sample and then incubated overnight on ice. The mixture was centrifuged and the supernatant kept for amino acid analysis. The amino acids were automatically derivatized with OrthoPhtalic Aldehyde (OPA) and 9-fluorenylmethyl-chloroformiate (FMOC-C1). The derivatives were separated on a Hypersil AA-ODS column (Agilent Technologies) at 40 °C with a linear gradient of acetate buffer (pH 7.2) with triethylamine (0.018%), tetrahydrofuran (0.3%) and acetonitrile. A diode array detector was used to detect OPA derivatives at 338 nm and FMOC derivatives at 262 nm.

### GAD activity measurements

For specific GAD activity measurements, 150 mg of cells were washed twice with 0.2% KCl (w/v) and suspended in 3 mL sodium acetate buffer (100 mM, pH 4.6) containing 4.5 mM MgCl_2_, 22% (v/v) glycerol, and 1.5 mM DTT. This mixture was divided into three tubes each containing 6 mg of glass beads. The cells were then lysed in a FastPrep-24 homogenizer (MP Biomedicals, Illkirch, France) using 6 cycles of 30 s at 6.5 m/s interrupted by 1 min incubation on ice. Cell debris were removed by centrifugation for 15 min at 10,000× *g* at 4 °C. The supernatant was used for enzyme assays, and the protein concentration in the extract was measured using the Bradford method. Enzyme assays were performed with 0.5 mL of substrate solution, consisting of 20 mM sodium glutamate and 2 mM pyridoxal phosphate (PLP) incubated at 30 °C then mixed with 0.5 mL supernatant. Samples (100 µL) were taken every 30 min until 4 h and inactivated by boiling for 5 min to stop the decarboxylation reaction. Reaction mixtures were subsequently analyzed for the presence of GABA using HPLC. One unit of enzyme activity was defined as the amount of enzyme that converted 1 mmol of glutamate per min and per g of protein.

### RNA extraction and gene expression analysis

A culture volume corresponding to 6 mg dry weight biomass was harvested and frozen immediately in liquid nitrogen. Before cell lysis, each sample was centrifuged (4 °C, 5 min, 4800 rpm), washed with 1 mL of TE buffer (Tris-HCl 10 mM, pH 8, EDTA 1 mM) and resuspended in 500 µL of TE buffer. Cells were disrupted at 4 °C (6.5 m/s, 4 cycles, 30 s, 1 min cooling on ice) on a FastPrep-24 (MP Biomedicals) with glass beads (0.6 g), 25 µL of SDS (20%), and 500 µL of phenol (pH 4.7). Cell debris and phenol were eliminated by centrifugation and (4 °C, 25 min, 13,000 rpm). RNA from the aqueous phase was extracted with RNeasy midi kit (Qiagen). The standard protocol (precipitation, washing and elution) including the DNase I treatment described in the manufacturer’s instructions. RNA concentration and quality were measured using a NanoDrop spectrophotometer and an Agilent Bioanalyzer (Santa Clara, CA, USA). The samples were subjected to reverse transcription using Super Script II reverse transcriptase (LifeTechnology), as previous described [39]. RT-qPCR was performed using a SYBR green-based detection protocol (Life Technology) with an Opticon 2 real-time PCR detection system (Bio-Rad) and MyIQ software (Bio-Rad). Primers (Table 1) specificity and PCR efficiency were analyzed on genomic DNA range prior to quantification. The *tuf* gene was used as internal standards for normalization. Variations of gene expression between strains were calculated with NCDO2727 (condition without chloride) as reference with the ΔΔCt method [40] and expressed as fold changes (FC).

**Table 1:** Sequences of primers used for quantitative RT-PCR.

Gene name	Forward Primer	Reverse Primer
*gadB*	TGGGAAAAATTCTGTGTTTATTGGGATATTGAAATG	CCAAAGCTTTGATATCATCATAACGACCAG
*gadC*	CGCTTCAATGGTTTTGACTGTCTATGAG	TTCAACCGTCGCCATTTCTGC
*gadR*	GACCGTTCAAATATATCTAGATTTGAACATGGAAA	CCAATTCTAATATAAAAAATTCTTGGAAATTCGGC
*tuf*	AAGGAGTGGTTTGTCAGTGTCG	CTTGGTGCTTTGAACGGTGAAC

### Sequence analysis

All the genome sequences of the representative strains used in this study (except those of strains MG1388, S170, CIRM-BIA 1564 and EIP3I) were obtained from the NCBI database. For strains MG1388, S170, CIRM-BIA 1564 and EIP3I, genomic DNA was extracted using the GenElute Bacterial Genomic DNA kit (Sigma-Aldrich) according to manufacturer instructions. Short-read whole genome sequencing was performed on an Illumina MiSeq sequencer (Illumina, Inc.,San Diego, US-CA) using 2 × 150 bp paired end reads with 100x average coverage. The raw data were analyzed using tools from the GALAXY website (https://usegalaxy.eu/). Briefly, the reads were assembled to contigs using Unicycler (version 0.4.8.0) with bold assembly and default parameters. Contigs shorter than 100 bp were excluded.

The GAD cluster, a region of approximately 4200 bp encompassing the *gadR*, *gadC*, *gadB* genes and their specific promoters, was extracted from the sequences of the representative strains (21 *L. lactis* strains, 8 *L. cremoris* strains with the lactis phenotype and 7 *L. cremoris* strains with the cremoris phenotype) using BLAST software (https://blast.ncbi.nlm.nih.gov/Blast.cgi). The nucleotide sequences were then aligned using the ClustalW algorithm with the software MEGA X (https://www.megasoftware.net/home). All raw and processed sequencing data generated in this study have been submitted to the NCBI BioProject database under accession number PRJNA960850.

## Results

### Similar organization of GAD system in different lactococci

**Fig. 1:**
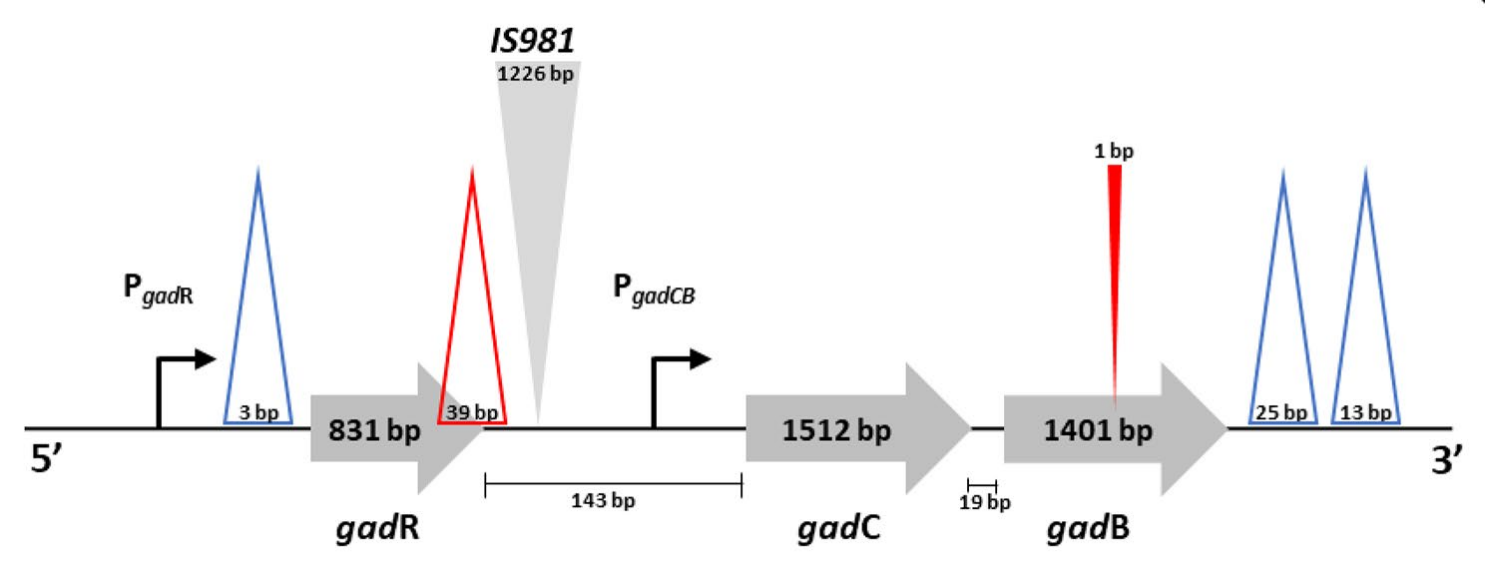
Schematic representation of genetic organization of the GAD genes (i.e. regulator *gadR*, glutamate/GABA antiporter *gadC*, glutamate decarboxylase *gadB*, and two promoters P_*gadR*_ and P_*gadCB*_) in lactococci: **∆** (blue) deletions observed in all *L. cremoris* species relative to *L. lactis*, **∆** (red) deletion or ▼ (red) insertion only observed in *L. cremoris* species with the cremoris phenotype, ▼ (grey) insertion site for the IS*981* element in *L. lactis* S642. (not drawn to scale)

We investigated the genetic organization of the GAD system by nucleotide sequence alignment in 36 lactococci, including 21 *L. lactis* and 15 *L. cremoris* species with an equal number of lactis and cremoris phenotypes for the latter (Additional file 1 Table S1). In all but one of the studied strains, the GAD system was found to be organized in the same way as described by Sanders et al. [37] for *L. lactis* MG1363, namely with a positive regulator encoded by *gadR* and expressed from the P_*gadR*_ promoter and the *gadCB* operon, consisting of *gadC* (encoding glutamate/GABA antiporter) and *gadB* (encoding GAD), located downstream of the P_*gadCB*_ promoter, ensuring rapid co-regulation of GAD and the transporter (Fig. 1).

The one exception was *L. lactis* S642, which has a 1226 bp insertion sequence element (IS*981*) in the P_*gadCB*_ promoter between the − 10 and − 35 boxes (Fig. 1). This insertion sequence introduces a new − 35 box, leading to a “hybrid” P_*gadCB*_ promoter with the − 10 box of native P_*gadCB*_.

### Differences in GAD genes between *L. lactis* and *L. cremoris* species

**Fig. 2:**
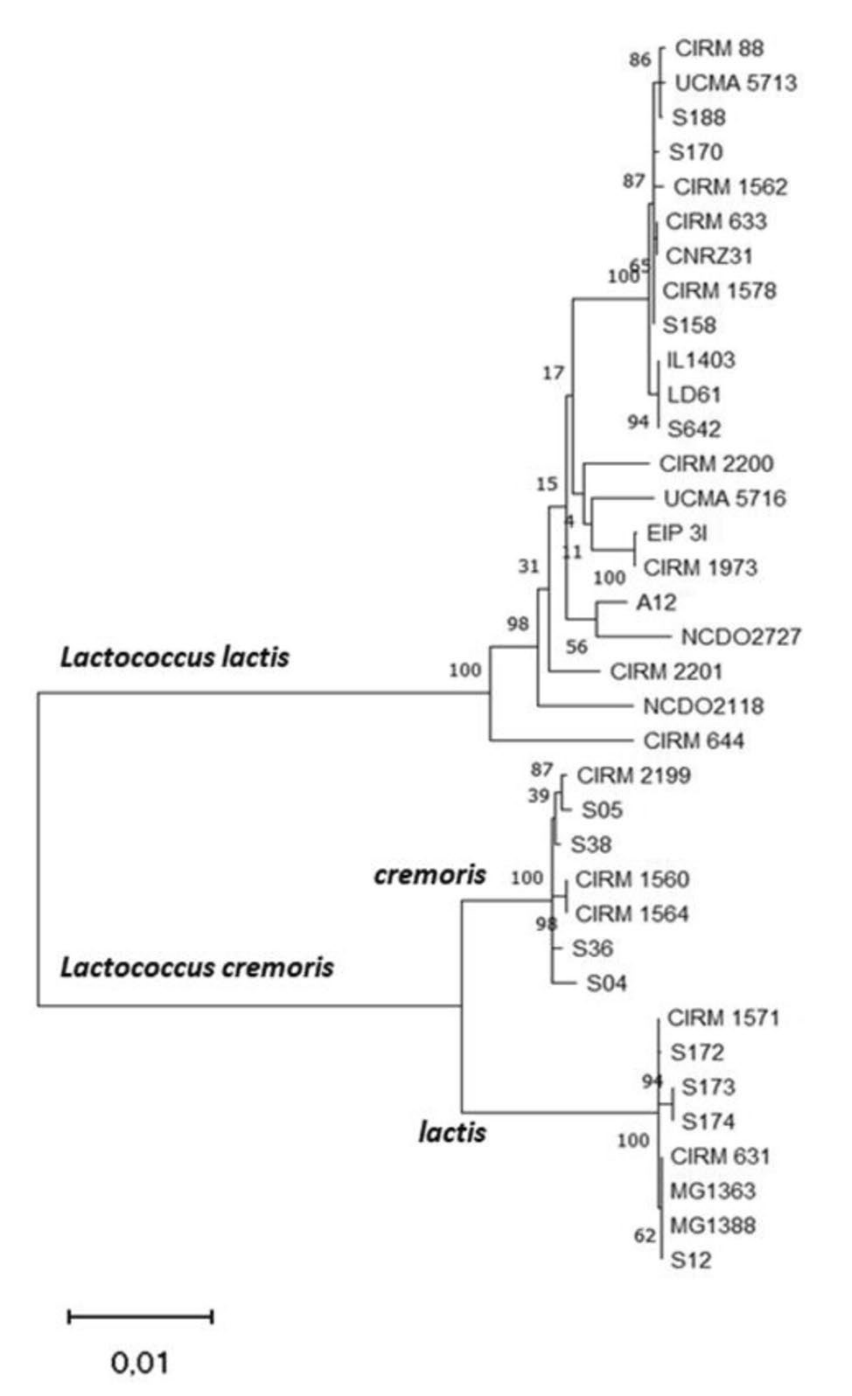
Phylogenetic tree based on GAD system gene sequences. The tree was generated with the software MEGAX using the maximum likelihood method and the *Tamura 3-parameter* model with 1,000 bootstraps. The numbers at the branches indicate supporting bootstrap values. The scale bar indicates 0.01 substitution per nucleotide position

The phylogenetic tree built from the alignment of nucleotide sequences of GAD system genes distinctly highlights the two species, *L. lactis* and *L. cremoris*, and also separates the lactis and cremoris phenotypes of *L. cremoris* strains (Fig. 2). It is remarkable that this phylogenetic tree based only on a comparison of GAD system nucleotide sequences captures the genotype/phenotype disparities within the *Lactococcus* genus. These results are consistent with those obtained by comparing the entire genomes of *L. lactis* and *L. cremoris* species [29].

Several deletion regions (shown in Fig. 1) were found in the sequences of the GAD genes. A 25-bp and a 13-bp deletion were observed in all *L. cremoris* species in the 3’ untranslated region of the *gadCB* operon. These deletions, described previously by Nomura et al. [41], were used to distinguish the two phylogenic groups corresponding to the *lactis* and *cremoris* species. Our in-depth analysis of the nucleotide sequences revealed two additional deletions; first, a 3-bp deletion in the P_*gadR*_ promoter specific to *cremoris* species; and second, a 39-bp deletion specific to *L. cremoris* with the cremoris phenotype. This deletion extends 13 bp upstream of the *gadR* stop codon to the inverted repeats characteristic of the end of the *gadR* transcript. Since this large deletion has never previously been detected, we confirmed its presence by PCR analysis of seventeen *L. cremoris* strains with the cremoris phenotype whose sequence is unknown (Additional file 2 Fig. S1). Finally, a single thymine insertion was observed only in *L. cremoris* with the cremoris phenotype. This insertion, reported previously by Nomura et al. [37], is located in the *gadB* gene and leads to the formation of a stop codon, producing a truncated GadB protein 56 amino acids shorter than in the other strains.

### High and variable levels of GABA production in *L. lactis*

**Fig. 3:**
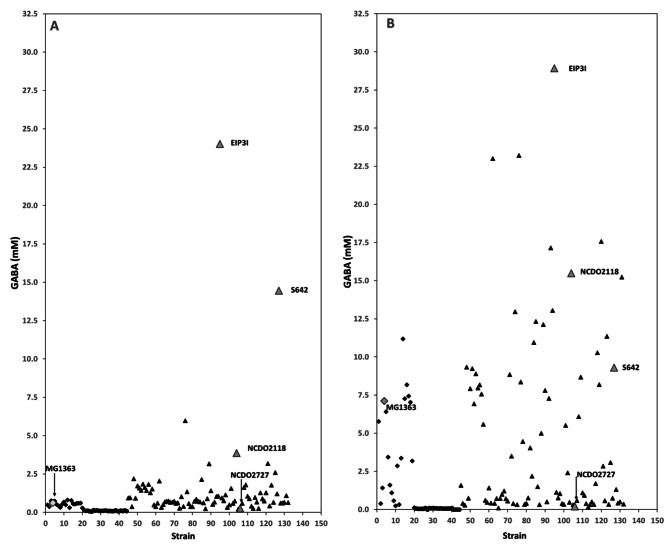
GABA production (mM) of 132 lactococci (▲ *L. lactis*, ♦ *L. cremoris* phenotype lactis, ● *L. cremoris* phenotype cremoris) after 24 h of growth on glucose-YE medium supplemented with 34 mM L-glutamic acid without NaCl (**A**) and with 0.3 M NaCl (**B**). Values represent the mean of duplicate experiments. The strains mentioned in the text are MG1363, a *L. cremoris* strain with a lactis phenotype ♦, and NCDO2118, NCDO2727, S642 and EIP3I, which are *L. lactis* strains▲

The diversity of GABA production was studied in 132 *Lactococcus* strains (the 36 sequenced strains described above and 96 news strains) covering all the different groups (88 *L. lactis* strains, 20 *L. cremoris* strains with the lactis phenotype and 24 *L. cremoris* strains with the cremoris phenotype). Bacterial cells were grown in duplicate in glucose-YE medium supplemented with L-glutamic acid (the precursor for GABA production). Overall, a great diversity of production was achieved with a highly strain-dependent GABA production ranging from 0 to 24 mM between the different lactococci under study (Fig. 3A). This diversity in GABA production was apparently unrelated to the niche of origin of the strains (Additional file 1 Table S1).

The strains that did not produce GABA are all of *L. cremoris* species with the cremoris phenotype. These strains all have the following phenotypic characteristics: no growth at 40 °C or in 4% (w/v) NaCl and inability to deaminate arginine (Additional file 1 Table S1). These phenotypic characteristics were never found together in any of the *L. lactis* strains or in any of the *L. cremoris* strains with a lactis phenotype, which produced very little GABA (Additional file 1 Table S1).

GABA production in the *L. cremoris* strains with a lactis phenotype ranged from 0.3 to 0.8 mM (0.55 ± 0.16 mM on average), demonstrating that GABA production varies little in this group. The MG1363 strain (highlighted in Fig. 3A), which is often used as a model strain in the literature, does not appear to be a good GABA producer as it only yielded 0.62 mM GABA. Although a majority (61%) of *L. lactis* strains produced GABA at similar concentrations (0.59 ± 0.21 mM) as those produced by *L. cremoris* with the phenotype lactis, several *L. lactis* strains were found to produce between 1 and 2.5 mM GABA and four *L. lactis* strains produced GABA at more than 2.5 mM, notably *L. lactis* NCDO2118, (highlighted in Fig. 3A) which produced 3.85 mM GABA. These production levels were greatly exceeded by two other *L. lactis* strains: *L. lactis* S642 and *L. lactis* EIP3I, which respectively yielded 14 and 24 mM GABA (Fig. 3A). These values were unexpected based on the literature and illustrate the great variability of GABA production in lactococci and particularly among *L. lactis* strains.

### Strain-dependent and variable effect of chloride on GABA production

Chloride ions are considered to be the most important factor for GABA production in lactococci [42]. We therefore investigated the existence and magnitude of any chloride-mediated increase in GABA production among the 132 studied strains, by growing them in the same conditions in the presence of 0.3 M NaCl.

The increase in GABA production in the presence of chloride varied considerably between strains (Fig. 3B). For some strains, no increase in GABA production was observed. In particular, *L. cremoris* strains with the phenotype cremoris remained unable to produce GABA (Fig. 3B). Similarly, the low levels of GABA production observed for some *L. lactis* strains remained unchanged in the presence of chloride; for instance, the NCDO2727 strain highlighted in Fig. 3 produced the same low level of GABA (about 0.2 mM) with and without NaCl. For a small number of lactococci, an unexpected decrease in GABA production was even observed. *L. lactis* S642 for example, which stood out by its high production capacity of 14.4 mM in the absence of chloride, produced just 9.3 mM GABA in the presence of chloride (Fig. 3B). However, for the large majority of lactococci, GABA production was higher in the presence of chloride, but with highly variable increases ranging from 1.2 to 16-fold depending on the strain. For example, in the presence of chloride, *L. lactis* NCDO2118 and *L. cremoris* MG1363 with a lactis phenotype (Fig. 3B) respectively produced 4 and 12 times more GABA than without chloride (15 and 7 mM versus 3.8 and 0.6 mM, respectively). Although the overall increase in GABA production was greater for *L. lactis* strains (1.1 to 28 mM) than for *L. cremoris* with the lactis phenotype (1.1 to 11 mM), the variability of the effect of chloride was similar for both groups, with increases ranging in both cases from 1.2- to 16-fold (Additional file 1 Table S1).

As adding chloride impacts growth rates, we evaluated whether this could have also affected GABA production. Growth rates varied from 0.24 to 1.14 h^-1^ without chloride and from 0.10 to 0.75 h^-1^ with chloride. However, levels of GABA production were not correlated to growth rate as shown by the scatter plots in the presence and absence of chloride (Additional file 3 Fig. S2). Thus, growth rate variability was not a major contributing factor to the diversity in GABA production of the studied lactococci.

### High variability in GAD activity and expression level of GAD genes

To go further in the understanding of this GABA production variability, we studied the potential relationship between GABA production and the specific activity of the GAD enzyme or the expression level of GAD genes. GAD activity measured in vitro under optimum culture conditions (i.e., maximum activity) reveals the expression level of the protein encoded by the *gadB* gene. This study was carried out on a limited panel of five strains selected to reflect the profiles highlighted above, namely four *L. lactis* strains (S642, EIP3I, NCDO2118, NCDO2727) and one *L. cremoris* strain with the lactis phenotype (MG1363), with markedly different levels of GABA production with and without NaCl (as shown in Fig. 3A/B). GAD specific activity varied considerably between strains and between culture conditions (Table 2). In the absence of chloride, GAD activities varied from 0.8 to 132 mmol/min/g protein and reached as high as 1464 mmol/min/g protein in the presence of chloride. Thus, the effect of chloride is also variable. GAD activity increased by a factor of 11, 43 and 45 in the EIP3I, NCDO2118, and MG1363 strains, respectively (Table 2), but remained constant in the NCDO2727 strain.

**Fig. 4:**
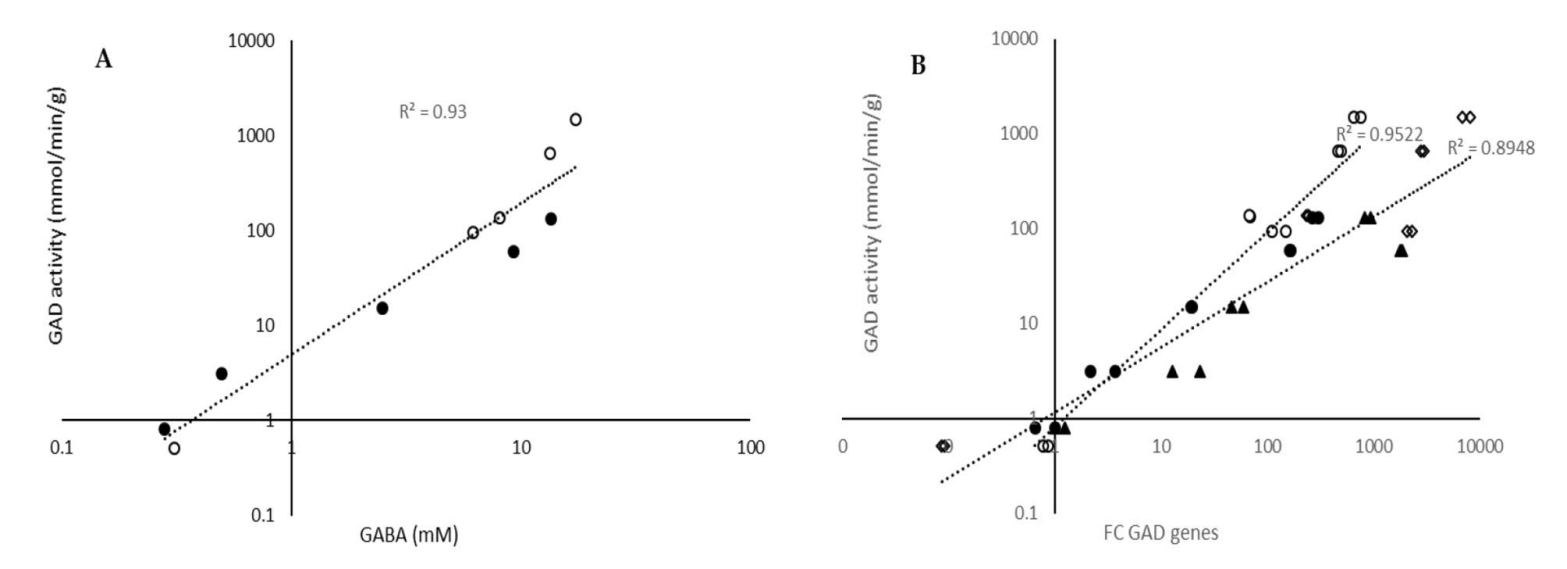
GABA production (mM) after 24 h of growth on glucose-YE medium supplemented with 34 mM L-glutamic acid for five strains without NaCl (filled symbol) and with 0.3 M NaCl (empty symbol) compared to specific GAD activity (mmol/min/g protein) (A) and specific GAD activity compared to gene expression fold change (FC) of *gadC* ∆▲ and *gadB* ○● (B)

At the same time, GABA production varied widely, from 0.3 to a maximum of 17.5 mM. Under all these conditions and media and in all these strains, GAD activity was well correlated with GABA production (Fig. 4A), indicating that cellular GAD activity is a key determinant of GABA production. To go further understand the regulation of GABA production regulation, we measured the expression of the genes in the GAD system (Additional file 4). GAD activity correlated well with the expression of the *gad CB* operon (*gadB* gene encoding glutamate decarboxylase and *gadC* gene encoding glutamate/GABA antiporter) (Fig. 4B). On the other hand, no link was observed between the expression of *gadR*, the regulator of the GAD system, and GAD activity.

**Table 2:** GAD activity (mmol/min/g) in glucose-glutamate-YE supplemented medium with and without NaCl (0.3 M) for five strains growth in a flask for 6 h. (n = 4)

NaCl	S642	EIP3I	NCDO2118	NCDO2727	MG1363
-	58.4 ± 2.9	131.9 ± 10.3	15.0 ± 2.8	0.8 ± 0.1	3.1 ± 0.7
+	93.1 ± 14.9	1464.3 ± 58.2	643.7 ± 104.4	0.5 ± 0.0	134.9 ± 29.5

## Discussion

The multiple health benefits of GABA have led to growing research interest. While GABA can be produced by chemical synthesis or by enzymatic biocatalysis [43, 44], studies have mainly focused on microbial fermentation [45–47]. Many strains of LAB can produce GABA, with *Lactobacillus* strains among the most efficient GABA producers described to date [48, 49]. Here, we studied the diversity of GABA production in lactococci to demonstrate that the *Lactococcus* genus is also an excellent candidate to synthesis great amounts of GABA.

The screening data obtained in this work highlights the wide range of GABA production levels in the panel of selected strains. However, the genetic organization of the GAD system was very similar in all studied lactococci bar one, *L. lactis* S642, where the presence of an IS*981* insertion sequence in the P_*gadCB*_ promoter moves the − 35 box away from the − 10 box. This IS*981* element creates a new − 35 box, leading to a “hybrid” P_*gadCB*_ promoter with the − 10 box of native P_*gadCB*,,_ which probably affects the transcription of the *gadCB* operon and hence GABA production by this strain. The effect of an IS*981* insertion on transcription has been described previously for other genes [50–53].

In spite of the similar genetic organization, GABA production varied greatly between the studied strains and was always zero for *L. cremoris* strains with the cremoris phenotype. Nucleotide sequence analysis revealed a specific 39-bp deletion for this lineage encompassing the 3’ end of the *gadR* gene and the transcription terminator. This probably affects *gadR*’s mRNA and as a result, the positive regulator GadR. Gong et al. [54] completely inactivated *gadR* in *Lactobacillus brevis* ATCC 367, leading to non-production of GABA through decreased expression of *gadC* and *gadB*. Furthermore, the insertion of a thymine into the *gad*B gene introduces a stop codon, shortening the GadB protein by 56 amino acids, and probably rendering it non-functional. Thus, the inability of *L. cremoris* strains with the cremoris phenotype to produce GABA, under all tested growing conditions, can be explained by altered GAD system sequences. These strains were also unable to produce ornithine from arginine. The two major pathways involved in resistance to acidic conditions are thus lacking in *L. cremoris* strains with the cremoris phenotype and this may explain the widely recognised strong acidifying properties of these strains.

A broad distribution of GABA production levels was also observed in the *L. cremoris* strains with a lactis phenotype, but GABA production varied more strongly and more widely in *L. lactis* than in *L. cremoris* species. Several genotypic observations may partly explain this difference. Firstly, a specific 3-bp deletion was observed in the P_*gadR*_ promoter in *cremoris* species. This deletion in the 5’UTR could reduce the efficiency of the P_*gadR*_ promoter relative to *L. lactis* strains and may thus lead to decreased *gadR* transcription and *gadCB* expression. This could contribute to the lower GABA production observed for *L. cremoris* with the lactis phenotype compared to *L. lactis*. Secondly, we identified a large number of single nucleotide polymorphisms (SNPs) in the GAD system genes in *L. lactis* strains. These SNPs do not involve active site residues and no correlation was found between these SNPs and GABA production. Thus, the genomic polymorphisms do not of themselves explain the diversity of GABA production observed in the *L. lactis* strains. On the other hand, the strong correlation between GABA production and cellular GAD activity, directly related to *gadB* gene expression, indicates that regulation of GAD gene expression is a major contributor to this diversity.

Finally, chloride ions were observed to have a strong effect on GABA production in lactococci. This effect has never previously been observed in LAB, whose *Lactobacillus* strains [49, 55]. Our results demonstrate the effect of chloride on the diversity of GABA production in lactococci. A majority of *L. lactis* and *L. cremoris* with the lactis phenotype produced more GABA (1.2 to 16 times more) in the presence of chloride. While it is well known that GadR is a positive regulator and that the presence of chloride leads to increased *gadCB* expression in strain MG1363 [35], the molecular mechanisms involved in this expression are not well understood in different *L. lactis* strains. A more detailed study of the regulation mechanism may provide an explanation for the difference in GAD activity and GABA production in the presence and absence of chloride in this species, and this deserves further attention. However, at the current state of knowledge, phenotypic screening is the best option to identify GABA overproducing strains with potential applications in the natural health product industry.

## Conclusions

In this study, combined genotype and phenotype analyses were used to reveal and understand the range of GABA production levels in *L. lactis* and *L. cremoris* species. Although all strains possess the *gadR*, *gadC* and *gadB* genes, this does not explain the observed diversity of GABA production, which has multiple causes. Indeed, while genomic polymorphisms may contribute to the non-production of GABA by *L. cremoris* with the cremoris phenotype, they do not explain the differences in production levels with and without chloride between *L. lactis* species. The regulatory mechanism of the GAD system that leads to these variations in cellular GAD activity is complex and requires further study.

The addition of chloride may reveal other GABA producers in *L. lactis* and in *L. cremoris* with the lactis phenotype species, which are low GABA producers in the absence of chloride.

Overall, these results provide new information on the diversity of GABA production in *Lactococcus* strains and highlight the richness of this bacterial species for the development of naturally fermented health products.

### Electronic supplementary material

Below is the link to the electronic supplementary material.

**Additional file 1:** Table S1. The 132 studied lactococci with their levels of GABA production, growth rates in media with and without chloride and ability to grow at 40 °C, in 4% (w/v) NaCl and to deaminate arginine.

**Additional file 2:** Figure S1. Strain differentiation based on the presence of a 39 bp deletion visualized by 3% agarose gel electrophoresis of PCR amplified products *. Lanes: 1, marker size 1 kb, 2, *L. cremoris* CIRM86, 3, *L. cremoris* CIRM1563, 4, *L. cremoris* S72, 5, *L. cremoris* S73, 6, *L. cremoris* S74, 7, *L. cremoris* S75, 8, *L. cremoris* S76, 9, distilled water, 10, *L. cremoris* S78, 11, *L. cremoris* S79, 12, *L. cremoris* S80, 13, *L. cremoris* S81, 14, *L. cremoris* S82, 15, *L. cremoris* S91, 16, *L. cremoris* S102, 17, *L. cremoris* S103, 18, *L. cremoris* S183, 19, *L. cremoris* S186, 20, *L. lactis* IL1403, 21, *L. cremoris* MG1363, 22, *L. lactis* NCDO2118, 23, reagent control with DNA, 24, marker size 100 bp.

**Additional file 3:** Figure S2. GABA production (mM) after 24 h of growth on glucose-YE medium supplemented with 34 mM L-glutamic acid without NaCl (A) and with 0.3 M NaCl (B) vs. the growth rates of the respective strains (µ in h^-1^).

**Additional file 4:** Table S2. Raw data for gene expression of *gadR*, *gadC*, *gadB* and gene control (tuf).

## Abbreviations

GABA: γ-aminobutyric acid
LAB: lactic acid bacteria
*L. lactis*: *Lactococcus lactis*
*L. cremoris*: *Lactococcus cremoris*
GAD: glutamate decarboxylase
SNP: single nucleotide polymorphism
HPLC: high performance liquid chromatography
PCR: polymerase chain reaction
