# Supplementary material for: Natural diversity of lactococci in γ-aminobutyric acid (GABA) production and genetic and phenotypic determinants
Source: Microb Cell Fact. 2023 Sep 9;22:178. doi: 10.1186/s12934-023-02181-4 (PMC10492284; doi:10.1186/s12934-023-02181-4)
Supplement: Supplementary file 2 — Additional file 2: Figure S1. Strain differentiation based on the presence of a 39 bp deletion visualized by 3% agarose gel electrophoresis of PCR amplified products *. Lanes: 1, marker size 1 kb, 2, L. cremoris CIRM86, 3, L. cremoris CIRM1563, 4, L. cremoris S72, 5, L. cremoris S73, 6, L. cremoris S74, 7, L. cremoris S75, 8, L. cremoris S76, 9, distilled water, 10, L. cremoris S78, 11, L. cremoris S79, 12, L. cremoris S80, 13, L. cremoris S81, 14, L. cremoris S82, 15, L. cremoris S91, 16, L. cremoris S102, 17, L. cremoris S103, 18, L. cremoris S183, 19, L. cremoris S186, 20, L. lactis IL1403, 21, L. cremoris MG1363, 22, L. lactis NCDO2118, 23, reagent control with DNA, 24, marker size 100 bp. [file 12934_2023_2181_MOESM2_ESM.docx]

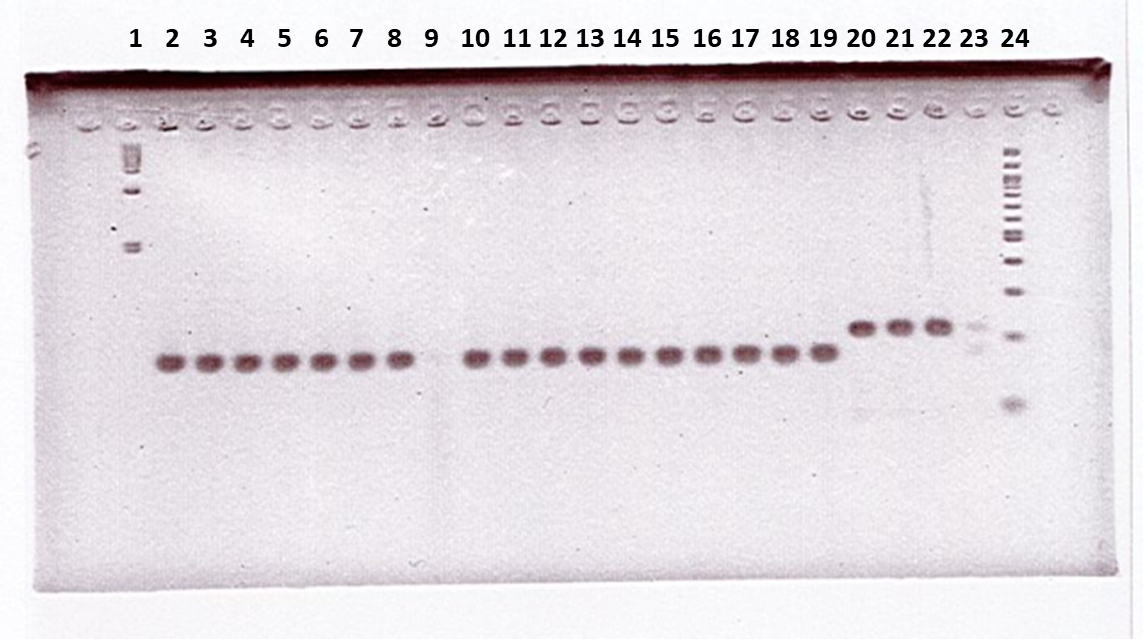


**Additional file 2 Figure S1.** Strain differentiation based on the presence of a 39 bp deletion visualized by 3% agarose gel electrophoresis of PCR amplified products. Strain differentiation based on the presence of a 39 bp deletion visualized by 3% agarose gel electrophoresis of PCR amplified products **.* Lanes: 1, marker size 1 kb, 2, *L. cremoris* CIRM86*,* 3, *L. cremoris* CIRM1563, 4, *L. cremoris* S72, 5, *L. cremoris* S73, 6, *L. cremoris* S74, 7, *L. cremoris* S75, 8, *L. cremoris* S76, 9, distilled water, 10, *L. cremoris* S78, 11, *L. cremoris* S79, 12, *L. cremoris* S80, 13, *L. cremoris* S81, 14, *L. cremoris* S82, 15, *L. cremoris* S91, 16, *L. cremoris* S102, 17, *L. cremoris* S103, 18, *L. cremoris* S183, 19, *L. cremoris* S186, 20, *L. lactis* IL1403, 21, *L. cremoris* MG1363, 22, *L. lactis* NCDO2118, 23, reagent control with DNA, 24, marker size 100 bp.

* The PCR amplification uses two specific designed primers from the gadR gene : forward primer 5-AGAATTTGGAGGAAAGAAGATAAAAG-3 and the reverse primer 5-TCATACCTCCTTATATTTATGATTGA -3. The PCR reaction mixtures contained 2 µL of each genomic DNA, 0.25 µL Q5 High-Fidelity DNA polymerase, 0.5 µL of each primer, 0.5 µL dNTPmix, 14.75 µl Q5reaction buffer in a total volume of 23 µl. The PCR conditions were : one cycle at 95°C for 5 min, 30 cycle at 95°Cfor 10 s, annealing 30 s at 59 °C and 72 °C for 2 min and a final extension cycle at 72 °C for 2 min. The PCR-amplified samples were analyzed by agarose gel electrophoresis by using a horizontal 3% (wt/vol) agarose gel. Gel weas run in 1 x TBE buffer at 100 V for 45 min then 50 V for 40 minutes.
