# Supplementary material for: Natural diversity of lactococci in γ-aminobutyric acid (GABA) production and genetic and phenotypic determinants
Source: Microb Cell Fact. 2023 Sep 9;22:178. doi: 10.1186/s12934-023-02181-4 (PMC10492284; doi:10.1186/s12934-023-02181-4)
Supplement: Supplementary file 3 — Additional file 3: Figure S2. GABA production (mM) after 24 h of growth on glucose-YE medium supplemented with 34 mM L-glutamic acid without NaCl (A) and with 0.3 M NaCl (B) vs. the growth rates of the respective strains (µ in h-1). [file 12934_2023_2181_MOESM3_ESM.docx]

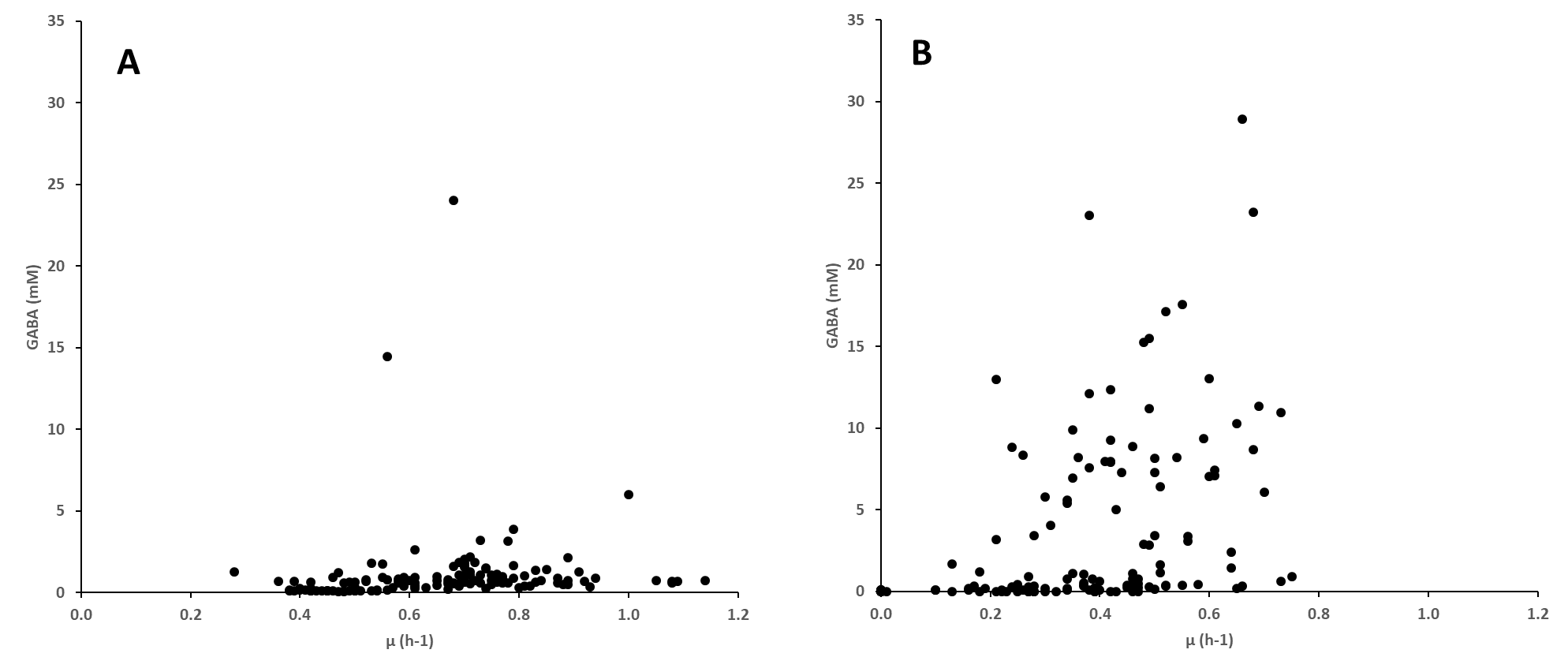


**Adiitional file 3 Figure S2.** GABA production (mM) after 24 h of growth on glucose-YE medium supplemented with 34 mM L-glutamic acid without NaCl (A) and with 0.3 M NaCl (B) *vs* the growth rates of the respective strains (µ in h^-1^).
